# Rapid Microwave-Assisted Chemical Recycling of Poly(*p*-Phenylene Terephthalamide)

**DOI:** 10.1021/jacs.4c17791

**Published:** 2025-02-21

**Authors:** Joël Benninga, Bert Gebben, Rudy Folkersma, Vincent S. D. Voet, Katja Loos

**Affiliations:** † Macromolecular Chemistry and New Polymeric Materials, Zernike Institute for Advanced Materials, University of Groningen, Nijenborgh 3, 9747 AG Groningen, The Netherlands; ‡ Circular Plastics, Academy Tech & Design, NHL Stenden University of Applied Sciences, Van Schaikweg 94, 7811 KL Emmen, The Netherlands; § Process Technology Department, Research and Innovation Center, Teijin Aramid BV, P.O. Box 5153, 6802 ED Arnhem, The Netherlands

## Abstract

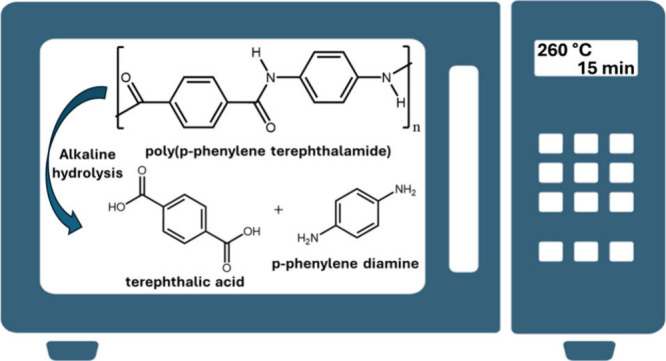

Back-to-monomer chemical recycling of polymers is crucial in achieving a circular plastics economy. Herein, we report the rapid microwave-assisted depolymerization of poly(*p*-phenylene terephthalamide) (PPTA), also known as Twaron or Kevlar. The alkaline hydrolysis of PPTA was conducted in a microwave reactor at temperatures ranging from 240 to 260 °C with reaction times of 1–15 min. The highest conversion (96%) was found after 15 min at 260 °C. The resulting monomers terephthalic acid and *p*-phenylenediamine were successfully purified (>99% purity) in good yields using extraction and precipitation methods. This work presents the fastest depolymerization of PPTA to date under relatively mild conditions, thereby encouraging a circular value chain for PPTA.

In 1965, DuPont research scientist Stephanie Kwolek discovered that poly(*p*-phenylene terephthalamide) (PPTA) could be dissolved into a liquid crystalline solution and spun into ultrahigh modulus aramid fibers, which are now known commercially as Kevlar or Twaron.^1^ Aramid fibers exhibit a significantly higher specific strength compared to steel, allowing them to provide equivalent strength at a reduced weight. In addition to their lightweight characteristics, aramids demonstrate extremely high crystallinity and ordering, along with remarkable thermal stability and chemical resistance, even in harsh environments. These superior properties have led to the widespread use of aramid fibers in various advanced applications, including ropes and cables, high-performance fabrics, advanced composites, and ballistic armor.^2−4^ Regrettably, these advantageous properties also pose significant challenges to the closed-loop recycling of aramid fibers. With a $2.9 billion market size,^5^ proper management of aramid fiber waste is crucial for both environmental and economic reasons. While postindustrial waste aramid fibers can undergo mechanical recycling, where they are chopped into pulp, the resulting materials typically exhibit relatively low economic value (i.e., downcycling) and are not recycled after their use.^6^ Additionally, the potential use of end-of-life aramid fibers in the development of high-value recycled composites is actively being explored. Loureiro et al. improved mechanical properties in a composite of nylon-6.6. reinforced with 10 wt % aramid fibers but faced poor fiber-matrix adhesion.^7^ Li et al. achieved a 62-fold increase in puncture resistance with 20 wt % Kevlar fibers in a nylon-polyester composite.^8^

Although using waste fibers to create composites is valuable, it is more sustainable to recycle them into new fibers as this reduces the demand for virgin materials. Fiber-to-fiber recycling can be realized through chemical recycling, which entails the depolymerization of polymeric waste (in the presence of a catalyst), converting it into monomers.^9^ These monomers can then undergo repolymerization to produce polymers of virgin-quality.^10−12^ Chemical depolymerization of PPTA has only been reported twice. Okajima et al. investigated the hydrolysis of PPTA (Kevlar) fibers into the constituent monomers *p*-phenylenediamine (PPD) and terephthalic acid (TPA).^13^ They reported the complete decomposition of the fibers and high monomer yields after 6 h of treatment with subcritical water and sodium hydroxide at 250 °C at 4 MPa. Treatment with subcritical and supercritical water without sodium hydroxide resulted in lower decomposition efficiencies and monomer yields. The same group reported the successful hydrolysis of a different *para*-aramid fiber called Technora, which is spun from copoly(*p*-phenylene-3,4′-oxidiphenylene terephthalamide), instead of from PPTA.^14^ Full decomposition of Technora fibers was achieved using subcritical water and sodium hydroxide at 250 °C and 4 MPa for 3 h, after which they obtained high yields of PPD, TPA and 3,4′-diaminodiphenyl ether.

Tian and co-workers reported the complete degradation of PPTA waste fibers after 6 h of treatment with 5 wt % NaOH in n-butanol at 180 °C.^15^ Interestingly, they claimed that the reaction mechanism is not an alcoholysis, and that they obtained PPD and TPA in high yields, instead of butanol-functionalized monomers. Given the long reaction times, high temperatures, use of organic solvents, and uneconomical purification steps for these reactions, it is evident that more research is required before large-scale chemical recycling of PPTA is feasible.

In this work, the microwave-assisted hydrolysis of PPTA without the use of organic solvents (Scheme 1) was investigated for the first time. Microwave-assisted depolymerization has gained growing attention in recent years and has been reported for various polymers,^16−18^ including poly(ethylene terephthalate) (PET),^19−21^ but had not yet been applied to the depolymerization of PPTA. While the microwave-assisted depolymerization of aliphatic polyamides has been reported previously,^16^ their properties are very different from PPTA. For instance, the intermolecular forces in PPTA are so strong that its melting point exceeds its decomposition temperature.^1^ Compared with conventional heating, microwave irradiation can substantially increase the rates of certain reactions and thus reduce reaction times and temperatures, while maintaining high chemo-selectivity and high yields.^22−24^ Additionally, depolymerization in the absence of organic solvents is gaining traction, which can potentially reduce the environmental impact associated with chemical recycling.^25−27^ Herein, we performed the microwave-assisted hydrolysis of PPTA at temperatures ranging from 240 to 260 °C and reaction times of 1, 5, and 15 min. The results demonstrate the rapid depolymerization of PPTA, followed by the efficient purification of terephthalic acid (TPA) and *p*-phenylenediamine (PPD), thus providing a cost-effective pathway for recycling waste PPTA into virgin-quality monomers, which can be repolymerized into high-grade PPTA.

**Scheme 1:**
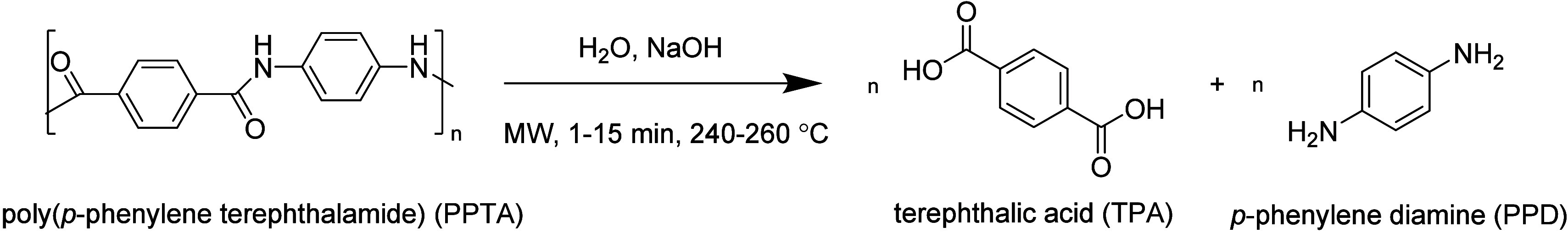
Microwave-Assisted Alkaline Hydrolysis Reaction of PPTA into the Monomers TPA and PPD

The microwave-assisted alkaline hydrolysis of PPTA powder under the specified conditions achieved high conversions (Figure 1), calculated based on the initial and final weights of PPTA in each reaction (see Supporting Information, Eq. S1). Given that PPTA does not dissolve in an alkaline aqueous environment, any filtration residue was assumed to be unreacted PPTA and insoluble PPTA oligomers, which is confirmed in the next section.^28^ The reaction conditions were chosen based on the restrictions of the microwave reactor, where 260 °C was the maximum operation temperature. Near-complete conversion (96%) was observed at 260 °C after 15 min, while reactions conducted at 240 and 250 °C exhibited minimal differences in conversion after the same duration. Even at 200 °C a conversion of 20% is found after 15 min (data not shown in Figure). The calculated energy consumption of the 260 °C, 15 min-reaction, and thus the energy required to reach near-complete conversion was approximately 1400 kJ. Significant depolymerization was observed even after 1 min at the investigated temperatures, with conversions only slightly lower than those achieved after 5 min at 240 and 250 °C. However, shorter reaction times exhibited greater variability in conversion, as evidenced by larger standard deviations compared to the 15 min reactions. This suggests that reactions conducted over shorter durations may not have reached equilibrium. Additionally, it is plausible that the distribution of microwave irradiation within the system is nonuniform. Although the reaction vessels rotate continuously, they remain stationary for approximately one second at the end of each 360-degree cycle, potentially causing some vessels to absorb more radiation than others, which may contribute to the observed variability in the conversions within this short 1 min period of time.^29,30^

**Figure 1:**
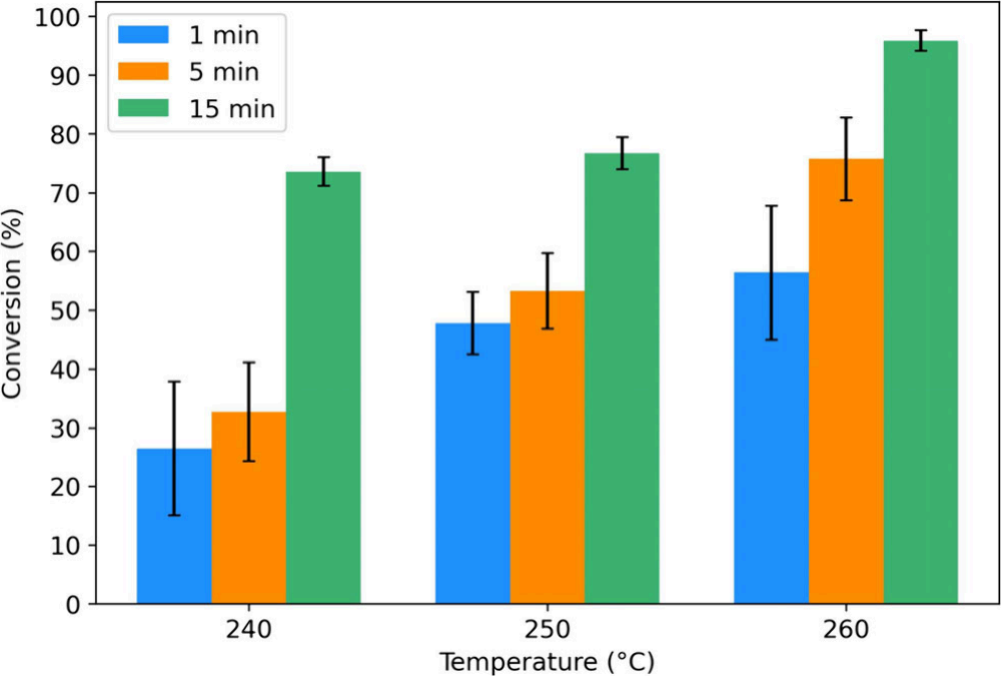
Conversion of PPTA after alkaline hydrolysis (2 M NaOH) in a microwave reactor under autogenous pressure. The experiments were conducted in triplicate, and the error bars show the deviation from the mean.

Fourier transform infrared spectroscopy (FTIR, Figure 2) confirmed that the filtration residue following hydrolysis was unreacted PPTA, as the FTIR spectra of PPTA (Figure 2a) and the residue (Figure 2b) were identical, verifying its composition. The vibrational modes corresponding to the amide functional group are present in both spectra. The N–H stretching mode can be seen at 3318 cm^–1^, while the strong C=O stretching vibration of the amide carbonyl is located at 1640 cm^–1^.

**Figure 2:**
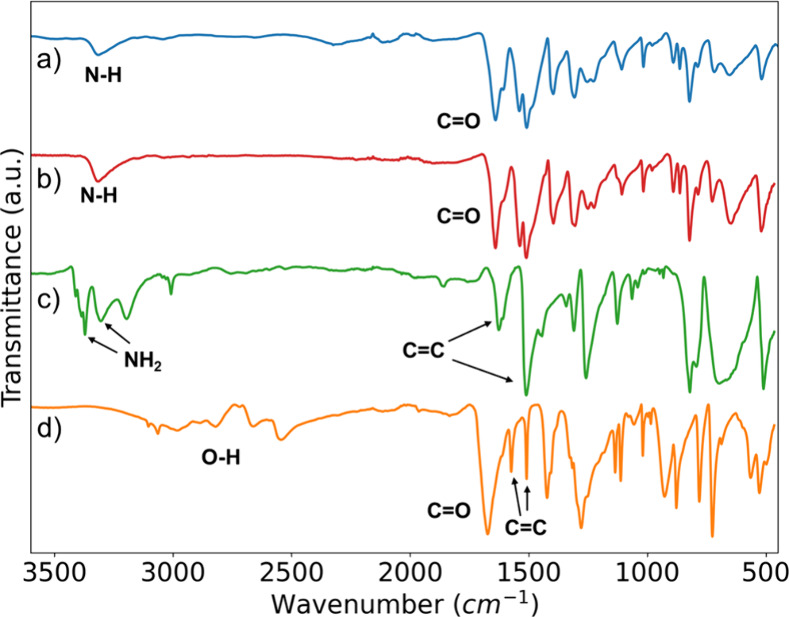
FTIR spectra of a) PPTA, b) residual PPTA after filtration of reaction mixture, c) purified PPD, and d) purified TPA.

^1^H NMR analysis (see Supporting Information, Figure S1) confirmed that the clear red filtrate contained dissolved PPD and disodium terephthalate (Na_2_TP), as terephthalic acid (TPA) exists in its deprotonated form in an alkaline environment. PPD was extracted using chloroform, and subsequent evaporation of the solvent yielded red PPD powder with a recovery rate of 76% (see Supporting Information, Eq. S2). The red color can be attributed to the fact that PPD is readily oxidized.^31^ The remaining Na_2_TP solution was acidified to pH 2 using hydrochloric acid, precipitating TPA. The precipitate was filtered and recrystallized with dimethylacetamide^32^ to form white needles of TPA with a yield of 65%. The formation of soluble oligomers^33^ may have distorted the monomer yields, as the conversion (Figure 1) used to calculate yields relies on the insolubility of PPTA. Traces of PPTA oligomer(s) were identified in the ^1^H NMR spectrum of the filtered reaction mixture (see Supporting Information, Figure S1). Yields can be improved using more sustainable and industrially relevant purification methods, such as the recrystallization of PPD^34^ and TPA^35^ directly in water, thereby reducing purification steps and eliminating the need for organic solvents. Currently, the E-factor (i.e., mass of waste/mass of product) of the unoptimized process was estimated to be 93 (see Supporting Information, Eq. S3), which is comparable to E-factors in the pharmaceutical industry.^36^ This result proves that our method is sustainable, and we can improve the E-factor by optimizing the workup process and increasing the PPTA loading.

The final products were characterized using FTIR. The FTIR spectra of PPD (Figure 2c) and TPA (Figure 2d) matched those of their corresponding commercial standards (see Supporting Information, Figure S2), confirming the identity of the recovered compounds. The characteristic peaks of PPD were found at 3371 and 3303 cm^–1^ for the stretching modes of NH_2_, and at 1626 and 1511 cm^–1^ for the C=C stretching modes of the benzene ring. The functional groups identified in the FTIR spectrum of TPA were the broad O–H stretching vibration (3200–2500 cm^–1^), the C=O stretching mode at 1674 cm^–1^, and the C=C stretching modes of the benzene ring (1574 and 1509 cm^–1^).

In addition, the products were characterized with proton and carbon nuclear magnetic resonance spectroscopy (^1^H NMR and ^13^C NMR, respectively). The ^1^H NMR spectrum of PPD (Figure 3a) displays two distinct signals, consistent with the expected structure of PPD. The prominent peak at 6.57 ppm is assigned to the four CH protons of the phenylene ring, while the peak at 3.33 ppm corresponds to the −NH_2_ functional groups. Similarly, two signals are seen in the ^13^C NMR spectrum (Figure 3b). The peak at 138.7 ppm corresponds to the carbons linked to the amino groups, whereas the peak at 116.9 ppm is attributed to the remaining CH carbons in the phenylene ring.

**Figure 3:**
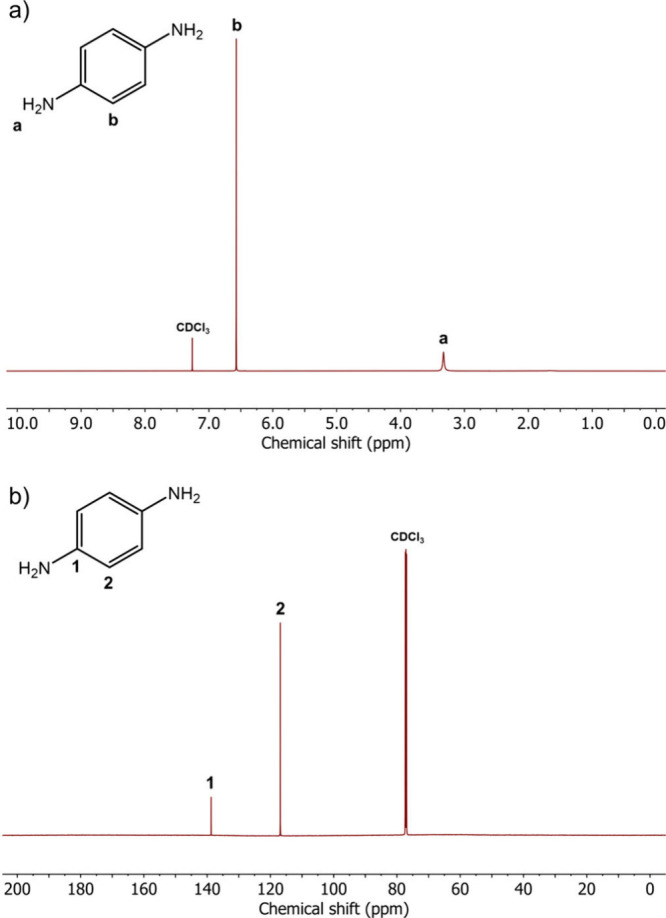
NMR spectra of PPD in deuterated chloroform (CDCl_3_). a) ^1^H NMR spectrum. b) ^13^C NMR spectrum.

The ^1^H NMR spectrum of TPA (Figure 4a) also clearly confirms the final product, indicating the presence of a highly deshielded proton at 13.29 ppm, which is ascribed to the −COOH protons in TPA. The strong signal at 8.04 ppm originates from the four CH protons in the phenylene ring. The small peak at 3.33 ppm is attributed to the presence of water in the hygroscopic NMR solvent. The ^13^C NMR spectrum (Figure 4b) reveals three signals at high chemical shifts. The most downfield peak at 166.7 ppm is ascribed to the −COOH carbons, while the signals at 134.4 and 129.5 ppm are attributed to the carbons linked to the −COOH groups and the CH carbons, respectively. The NMR spectra of both PPD and TPA align with literature data and represent highly pure monomer products of depolymerized PPTA.^15^ Furthermore, high-performance liquid chromatography (HPLC) was employed to determine that the purity of both monomers exceeded 99% (see Supporting Information, Figure S3).

**Figure 4:**
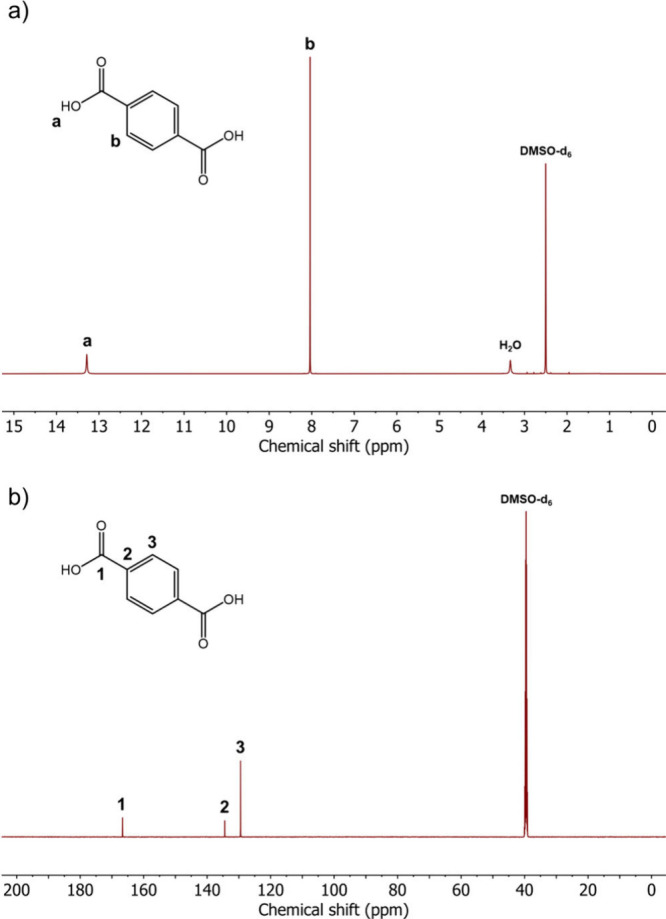
NMR spectra of TPA in deuterated dimethyl sulfoxide (DMSO-d_6_). a) ^1^H NMR spectrum. b) ^13^C NMR spectrum.

In conclusion, this study establishes the first-ever microwave-assisted depolymerization of PPTA and achieves the fastest depolymerization reported to date, reaching near-complete conversion at significantly lower temperatures than 300 °C. Specifically, 96% conversion was obtained after just 15 min of alkaline hydrolysis at 260 °C in a microwave reactor. This represents a significant improvement over the results of Okajima et al., who reported approximately 50% conversion at the same temperature after 20 min, and full conversion after 60 min using conventional alkaline hydrolysis on a similar scale.^13^ Our findings suggest a 4-fold increase in reaction rate.

This work clearly demonstrates the rapid depolymerization of high-production-volume PPTA under relatively mild conditions, presenting a highly promising pathway for closed-loop chemical recycling of PPTA at an industrial scale. The application of microwave reactors at such a scale is both feasible and advantageous, with microwave-heated processes being reported to exhibit over 30% greater energy efficiency than conventional methods.^37^ Additionally, scaling up microwave-driven processes has been shown to reduce specific energy consumption by 90–95%.^38^ To fully capitalize on this potential, future research should focus on improving monomer yields and developing more sustainable purification methods. Further experiments using PPTA fibers as starting material may also provide insights into optimizing depolymerization rates. The continued advancement of microwave-assisted chemical recycling has the potential to play a key role in establishing a fully circular aramid value chain, contributing to greater sustainability in the industry.

## Supporting Information Available

Experimental details, FTIR and NMR spectra, and HPLC chromatograms (PDF)
