# Supplementary material for: Rapid Microwave-Assisted Chemical Recycling of Poly(p-Phenylene Terephthalamide)
Source: J Am Chem Soc. 2025 Feb 21;147(9):7191–5. doi: 10.1021/jacs.4c17791 (PMC11887048; doi:10.1021/jacs.4c17791)
Supplement: Supplementary file 1 — ja4c17791_si_001.pdf [file ja4c17791_si_001.pdf]

Supporting Information

Rapid Microwave-Assisted Chemical Recycling of Poly(*p*-Phenylene Terephthalamide)

Joël Benninga [a,b], Bert Gebben [c], Rudy Folkersma [b], Vincent S. D. Voet [b], and Katja Loos [a*]

[a] *Macromolecular Chemistry and New Polymeric Materials, Zernike Institute for Advanced Materials, University of Groningen, Nijenborgh 3, 9747 AG Groningen, The Netherlands*
[b] *Circular Plastics, Academy Tech & Design, NHL Stenden University of Applied Sciences, Van Schaikweg 94, 7811 KL Emmen, The Netherlands*
[c] *Process Technology Department, Research and Innovation Center, Teijin Aramid BV, P.O. Box 5153, 6802 ED Arnhem, The Netherlands*
* Email: k.u.loos@rug.nl

Table of Contents

Experimental section 3

Materials 3

Milestone FlexiWAVE microwave reactor 3

Experimental details 3

FTIR spectroscopy 4

NMR spectroscopy 4

HPLC 4

^{1}H NMR spectrum of filtered reaction mixture 5

Figure S1. ^{1}H NMR spectrum of filtered reaction mixture in D_2O after microwave-assisted PPTA hydrolysis (250 °C, 5 minutes). 5

FTIR spectroscopy 6

Figure S2. FTIR spectra of a) commercial PPD. b) commercial TPA. 6

HPLC chromatograms 7

Figure S3. HLPC chromatograms of a) PPD. b) TPA. 7

Integrated and labeled NMR spectra of monomers 8

Figure S4. ^{1}H NMR spectrum of PPD in $CDCl_3$. 8

Figure S5. ^{13}C NMR spectrum of PPD in $CDCl_3$. 9

Figure S6. ^{1}H NMR spectrum of TPA in DMSO-d_6. 10

Figure S7. ^{13}C NMR spectrum of TPA in DMSO-d_6. 11

Experimental section

Materials

Poly(*p*-phenylene terephthalamide) (PPTA) containing trace amounts of *N*-methyl-2-pyrrolidone was kindly provided in powder form by Teijin Aramid BV. Sodium hydroxide pellets (analysis grade) were purchased from Boom BV. Chloroform (>99%), acetonitrile (>99.9%), chloroform-d ($CDCl_3$, 99.8 atom % D), deuterium oxide (D_2O, 99.9 atom % D, containing 0.75 wt% 3-(trimethylsilyl)propionic-*2,2,3,3-d*$_4$ acid (TSP-d_4)), dimethyl sulfoxide (DMSO, >99.9%), dimethyl sulfoxide-d_6 (DMSO-d_6, 99.5 atom % D), hydrochloric acid (37%), methanol (99.9%), *N,N*-dimethylacetamide (99%), *p*-phenylene diamine (>98%), terephthalic acid (>98%), and water with 0.1% trifluoroacetic acid (hypergrade) were purchased from Sigma-Aldrich.

Milestone FlexiWAVE microwave reactor

The experiments were conducted in a Milestone FlexiWAVE microwave reactor operating at a microwave frequency of 2.45 GHz. The maximum power is 1800 W, while the maximum reaction temperature is 260 °C, which is measured with an infrared sensor. The reactor can contain up to 15 100 mL Teflon vessels, and continuous stirring is possible with PTFE stirring bars. The minimum and maximum volume per vessel are 5 and 60 mL, respectively. In addition to polar solvents, non-polar solvents may be used but require adding microwave absorbing buttons. The maximum autogenous pressure per vessel is 100 bar.

Experimental details

Microwave experiments were conducted in a Milestone FlexiWAVE microwave reactor in triplicate by charging three separate 100 mL Teflon vessels with 1 g PPTA, 1.6 g NaOH, and 20 mL deionized water. The contents of the vessels were heated up to the desired temperature in 30 minutes under continuous stirring, and this temperature was maintained for 1, 5 or 15 minutes by irradiating with an average power of 500 Watt. Afterwards, the contents of the vessels were transferred to a beaker to which 80 mL deionized water was added to dissolve any excess *p*-phenylene diamine (PPD). The reaction mixture consisted of dissolved disodium terephthalate (Na_2TP), PPD, soluble oligomers, and insoluble unreacted PPTA (oligomers). The residual PPTA was removed by vacuum filtration, and subsequently dried overnight in a vacuum oven at 50 °C. The conversion was then calculated with Eq. S1:

$$Conversion\ (\%) = \frac{m_0 - m_1}{m_0} \cdot 100\% \qquad \text{(S1)}$$

where m_0 is the weight of PPTA before a reaction and m_1 is the weight of the dry PPTA residue.

PPD was extracted from the clear red filtrate using chloroform, which was subsequently evaporated to obtain red powder, which was finally dried overnight in a vacuum oven at 50 °C to obtain PPD with a yield of 76%. The remaining Na_2TP solution was acidified with hydrochloric acid to pH 2 to precipitate terephthalic acid (TPA). The precipitate was filtered, recrystallized with dimethylacetamide to form white needles, washed with deionized water, and finally dried overnight in a vacuum oven at 50 °C to obtain TPA with a yield of 65%. Yields were calculated with Eq. S2:

$$Yield\ (\%) = \frac{m_{product}}{m_{theoretical}} \cdot 100\% \qquad \text{(S2)}$$

where $m_{product}$ is the weight of the dried product and $m_{theoretical}$ is the maximum achievable weight based on conversion and the fact that 1 mole of PPTA (molar mass of repeating unit: 238.26 g/mol) should form 1 mole of TPA and 1 mole of PPD.

The E-factor was calculated with Eq. S3:

$$E\ factor = \frac{mass\ of\ waste}{mass\ of\ products} \tag{S3}$$

A single reaction was estimated to lead to 74 g of waste and 0.8 g of monomers. This does not include the dilution step after a reaction, which is only done for calculating conversions.

FTIR spectroscopy

FTIR spectra were recorded on a Thermo Scientific Summit FTIR spectrometer and were analyzed using Spectragryph software.

NMR spectroscopy

^{1}H NMR spectra were recorded on a Bruker CAB AV4 600 MHz basic spectrometer at room temperature (pulse width = 12 μs, spectral width = 12 MHz, relaxation delay = 1 s, 64 scans) using DMSO-d_6, $CDCl_3$, or D_2O as the solvent. ^{13}C NMR spectra were recorded using the same spectrometer at 151 MHz (pulse width = 10 μs, spectral width = 36 MHz, relaxation delay = 2 s, 1024 scans). The spectra were analyzed using MestReNova software.

HPLC

HPLC analysis was carried out using a Shimadzu LC-20 HPLC system equipped with PDA detection. LabSolutions software was used for peak integration. Chromatographic separation was achieved on a C18 column (250 × 4.6 mm, 5 μm). The column temperature was kept at 40 °C during chromatographic operation. For the analysis of PPD, samples and standards were dissolved in Milli-Q water and subsequently filtered with 0.2 μm filters (Sartorius). An injection volume of 10 μL was used and the samples were eluted with isocratic acetonitrile with a flow rate of 1 mL/min, while spectra were measured at 254 nm. For the analysis of TPA, samples and standards were dissolved in a mixture of Milli-Q and DMSO (60:40 v/v) and subsequently filtered with 0.2 μm filters. An injection volume of 2 μL was used and the samples were eluted with a gradient of methanol and water (0.1% trifluoroacetic acid) with a flow rate of 0.8 mL/min, while spectra were measured at 254 nm. The following gradient was used: 0-4 min, 10% methanol; 4-7 min, 30% methanol; 7-16 min, 60% methanol; 16-20 min, 10% methanol. Monomer purities were determined by extrapolating from the standard curve.

^{1}H NMR spectrum of filtered reaction mixture

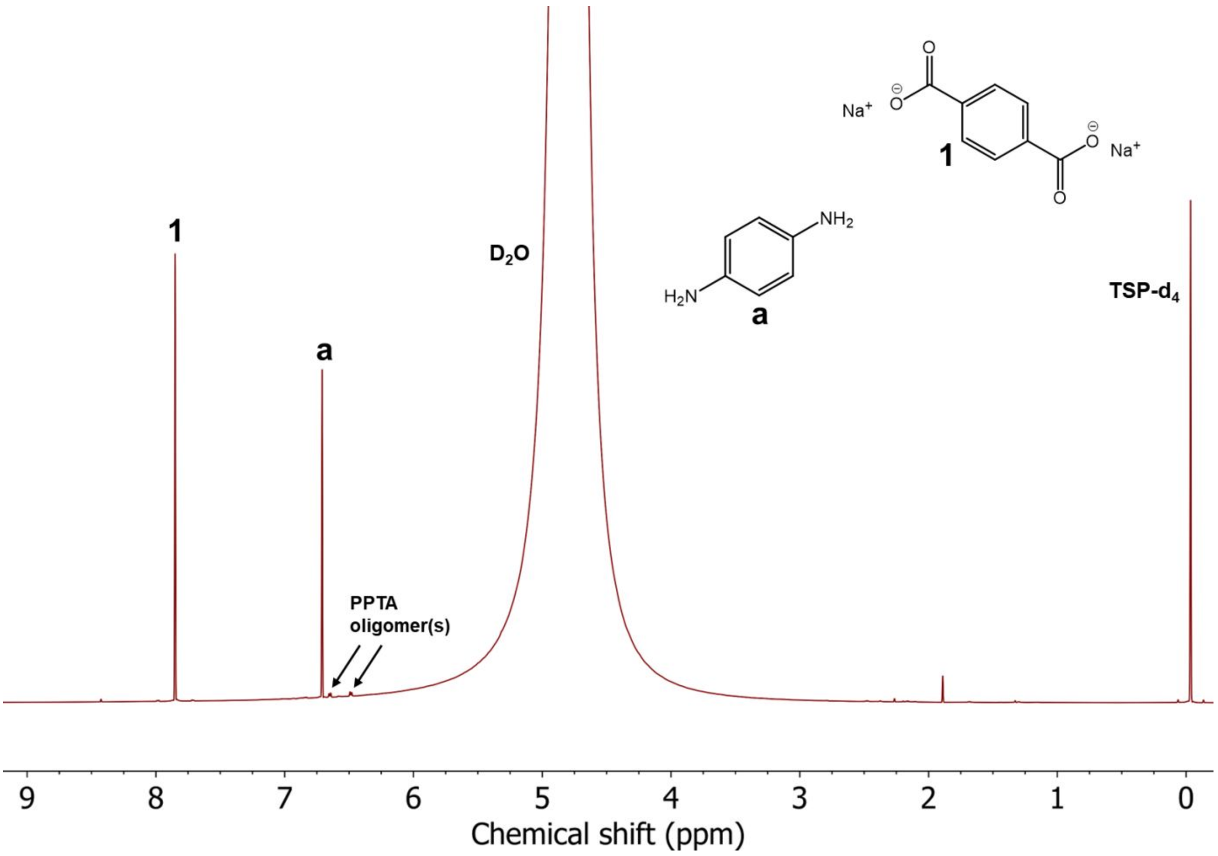

Figure S1. ^{1}H NMR spectrum of filtered reaction mixture in D_2O after microwave-assisted PPTA hydrolysis (250 °C, 5 minutes).

FTIR spectroscopy

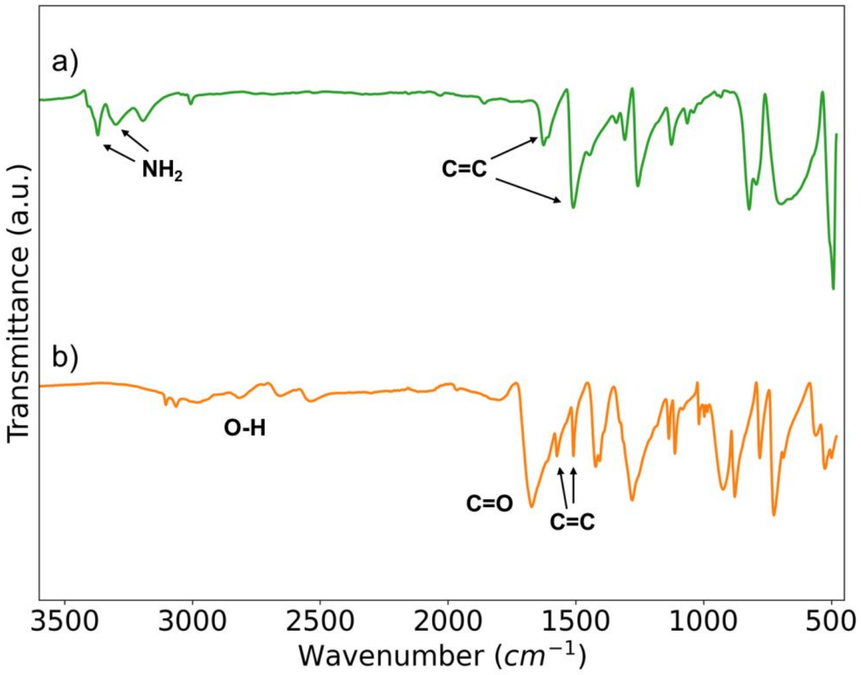

Figure 2

Figure S2. FTIR spectra of a) commercial PPD. b) commercial TPA.

Characteristic band frequencies

IR (PPTA, cm^{-1}): $\tilde{v}$=1640 (s) (C=O), 3318 (br) (N-H)

IR (PPD, cm^{-1}): $\tilde{v}$=1511 (s), 1626 (m) (C=C), 3303 (m), 3371 (m) (NH_2)

IR (TPA, cm^{-1}): $\tilde{v}$=1509 (m), 1574 (m) (C=C), 1674 (s) (C=O), 2500-3200 (br) (O-H)

HPLC chromatograms

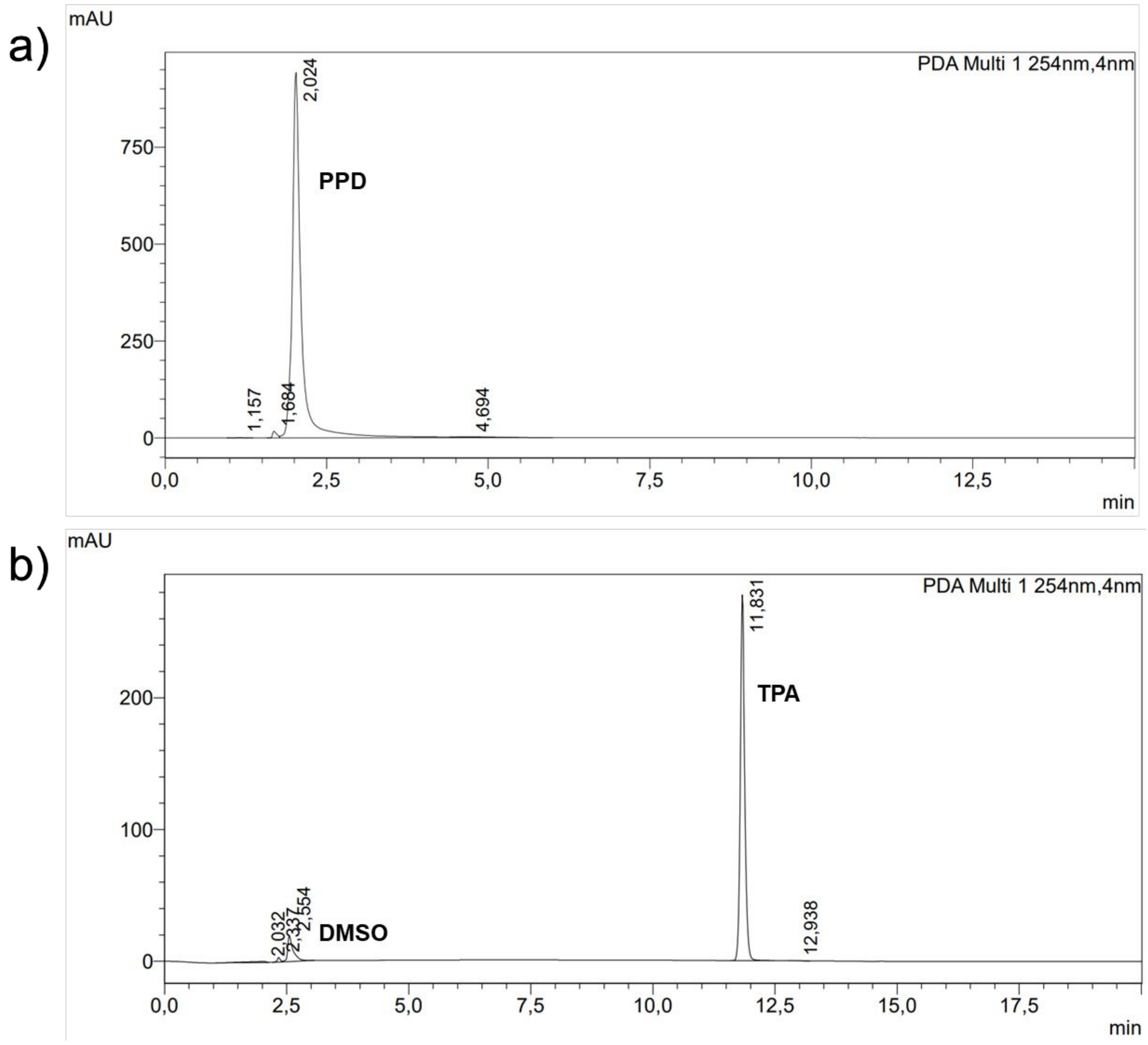

Figure S3. HLPC chromatograms of a) PPD. b) TPA.

Integrated and labeled NMR spectra of monomers

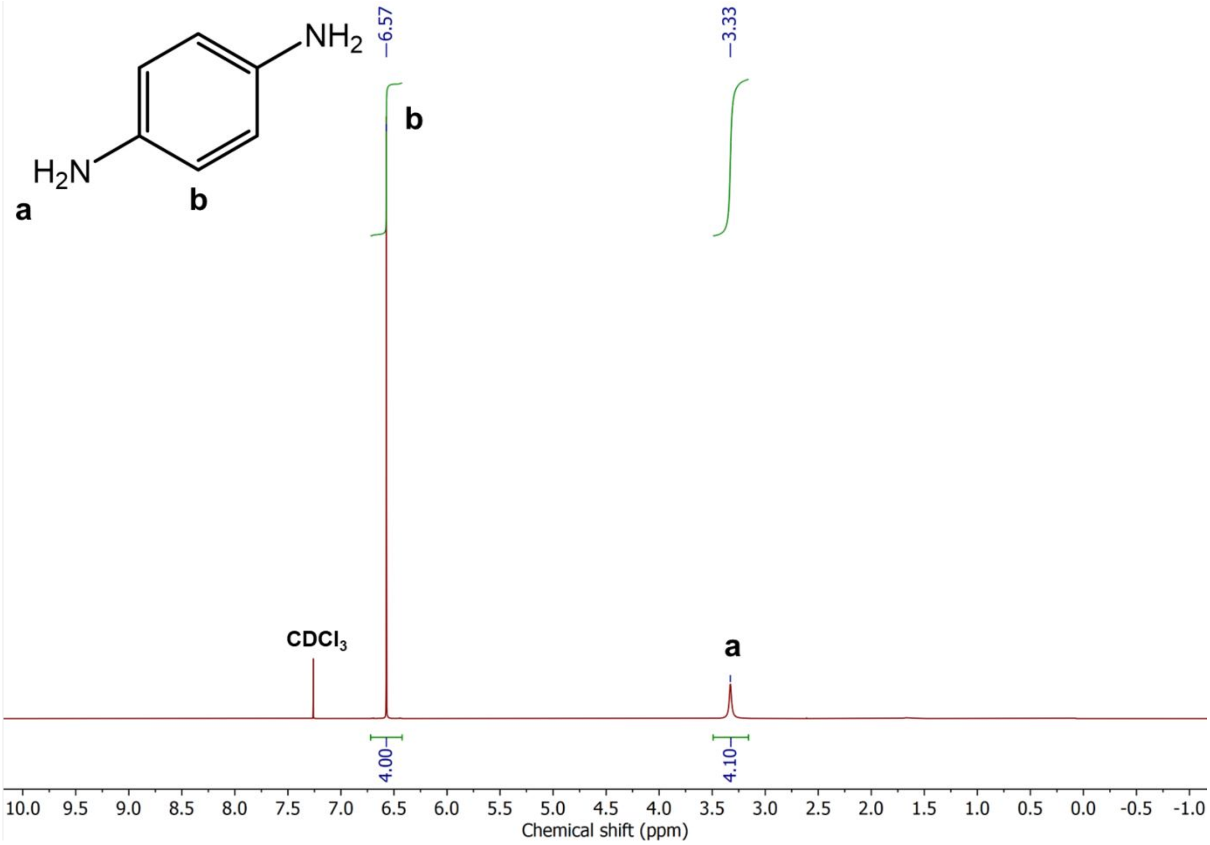

Figure S4. ^{1}H NMR spectrum of PPD in $CDCl_3$.

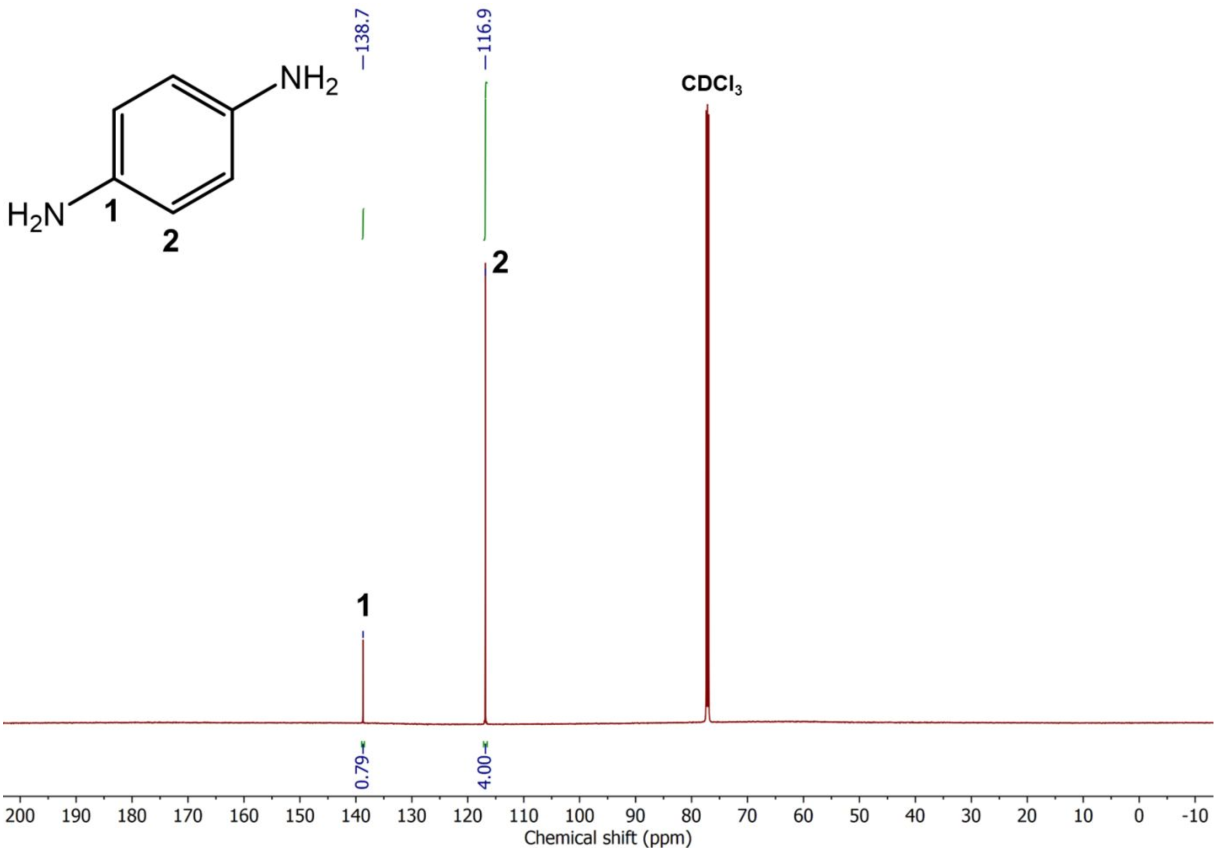

Figure S5. ^{13}C NMR spectrum of PPD in $CDCl_3$.

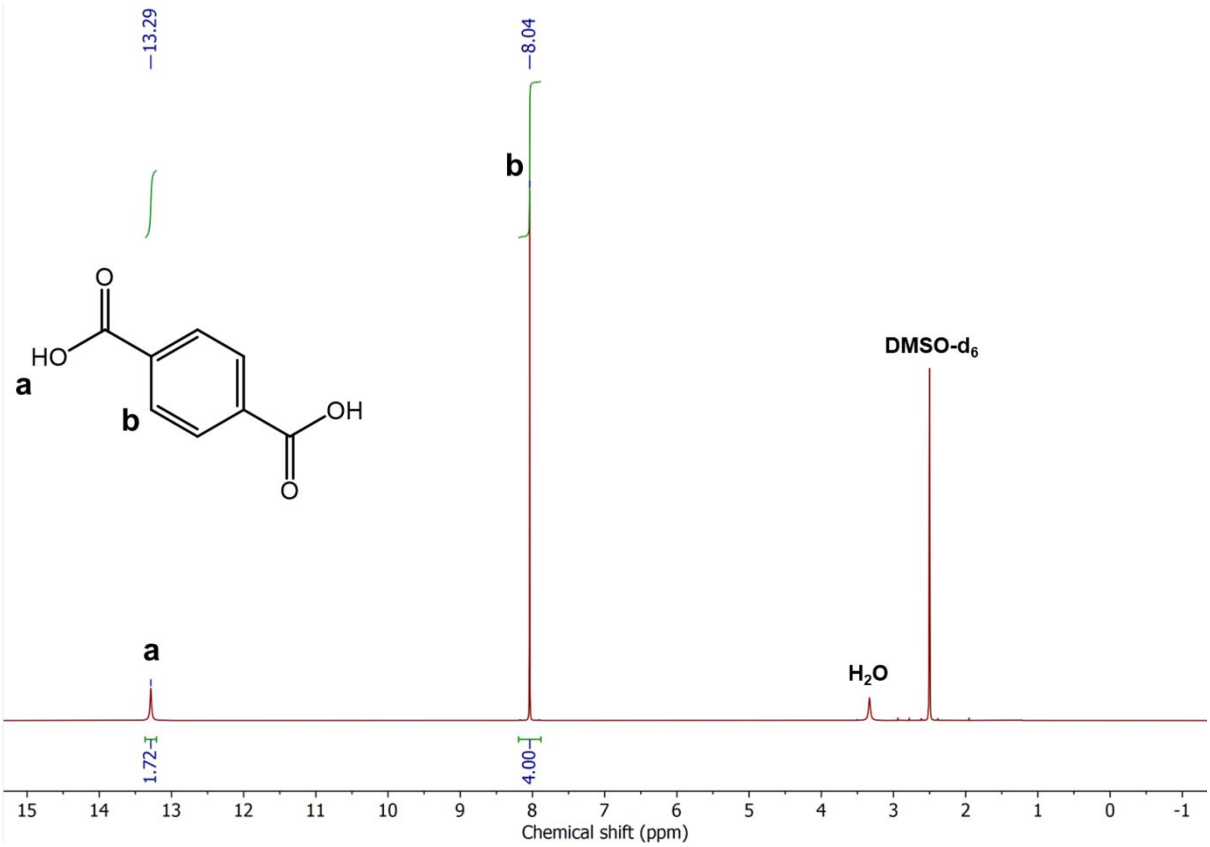

Figure S6. ^{1}H NMR spectrum of TPA in DMSO-d_6.

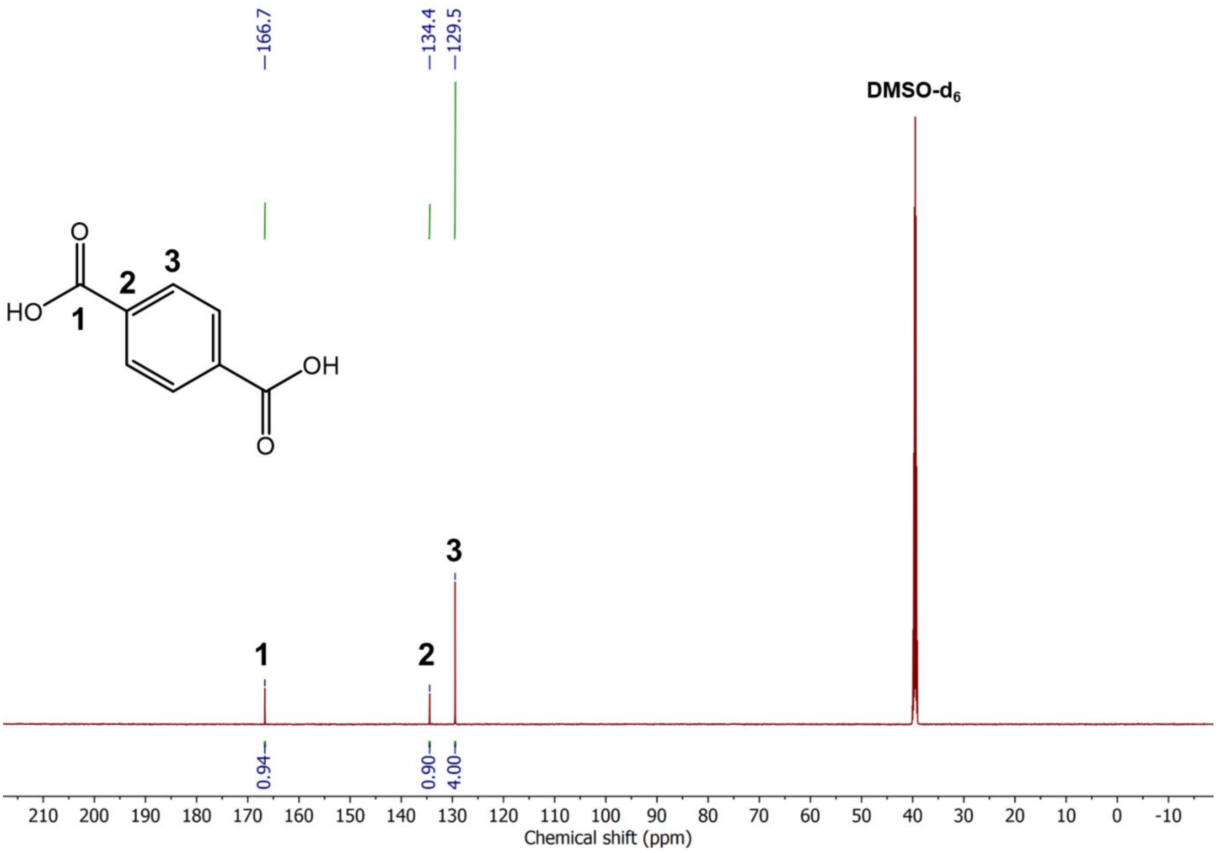

Figure S7. ^{13}C NMR spectrum of TPA in DMSO-d_6.
